# Supplementary material for: Metallic On-Chip Light Concentrators Fabricated by In Situ Plasmonic Etching Technique
Source: Nanomaterials (Basel). 2022 Nov 25;12(23):4195. doi: 10.3390/nano12234195 (PMC9739918; doi:10.3390/nano12234195)
Supplement: Supplementary file 1 [file nanomaterials-12-04195-s001.zip › nanomaterials-2039602-supplementary.pdf]

nanomaterials

MDPI

Supplementary Materials

Metallic On-Chip Light Concentrators Fabricated by In Situ Plasmonic Etching Technique

Lihua Cha [1] and Pan Li [2,3,4,*]

[1] School of Law, Central University of Finance and Economics, Beijing 100081, China
[2] Institute of Analysis and Testing, Beijing Academy of Science and Technology, Beijing 100089, China
[3] Department of Physics, Capital Normal University, Beijing 100048, China
[4] School of Information Technology, Beijing City University, Beijing 100083, China
* Correspondence: lipan@bcpca.ac.cn

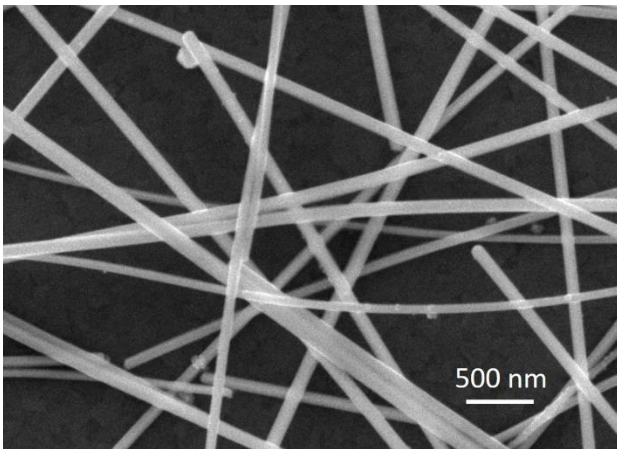

Figure S1. SEM image of the chemically synthesized Ag NWs with diameters around 100 nm. The NWs were prepared using a solution-phase polyol method [1].

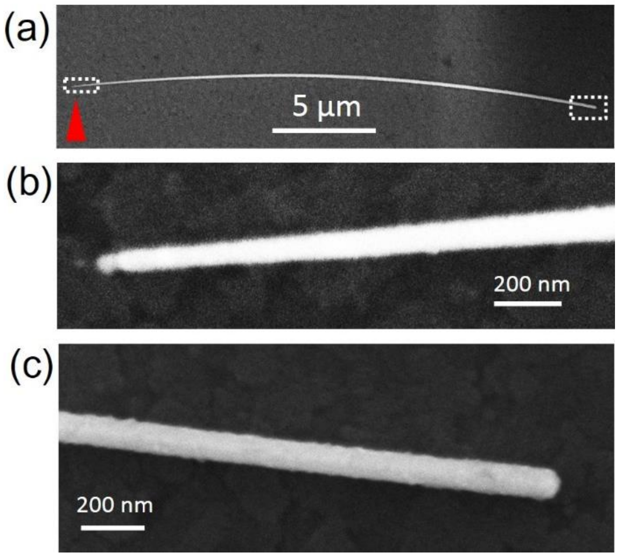

Figure S2. SEM images of a fused Ag nanowire fabricated by the etching on ZnO coated ITO substrate. (**a**) SEM image of the fused nanowire as a nanoneedle. The laser is focused on left part of the nanowire, marked by red arrow tip. The nanowire is fused after 70 s illuminating with laser power 200 mW. (**b**), (**c**) Zoom in images of the ends of the nanoneedle, and show a tip structure around 40 nm at the illuminated area. The diameter of the nanowire before etching can be found around 120 nm, as shown in the other end of the nanoneedle in (**c**).

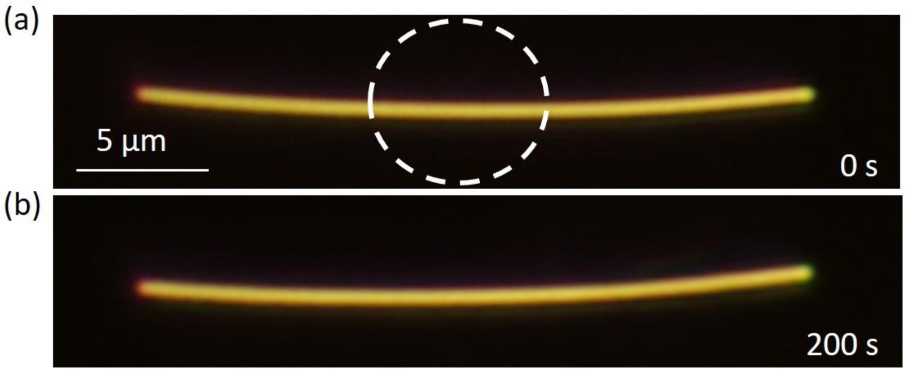

Figure S3. Dark field images of NW etching on glass with laser illumination time (**a**) 0 s, (**b**) 200 s. The white dash circle indicates the laser spot. The laser power is 200 mW, and the polarization is vertical to the long axis of the NW.

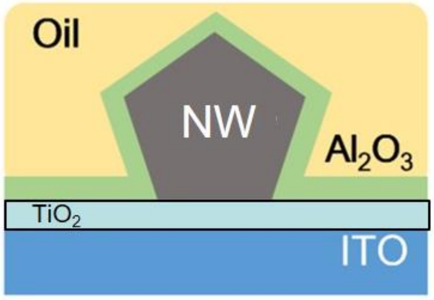

Figure S4. Schematic cross-section of the modelled NW on substrate covered by a layer of 10 nm Al_2O_3.

Reference

1. Sun, Y. G.; Xia, Y. N. Large-scale synthesis of uniform silver nanowires through a soft, self-seeding, polyol process, *Adv. Mater.* **2002**, *14*, 833-837.
